# Age, sex and graft choice are associated with the achievement of a patient acceptable symptom state five years after primary ACL reconstruction

**DOI:** 10.1002/ksa.70116

**Published:** 2025-10-27

**Authors:** Firathan Koca, Christoffer von Essen, Eric Hamrin Senorski, Kristian Samuelsson, Anders Stålman, Riccardo Cristiani

**Affiliations:** ^1^ Department of Molecular Medicine and Surgery, Section of Sports Medicine Karolinska Institutet Stockholm Sweden; ^2^ Stockholm Sports Trauma Research Center (SSTRC), FIFA Medical Centre of Excellence Sophiahemmet Stockholm Sweden; ^3^ Department of Health and Rehabilitation, Institute of Neuroscience and Physiology, Sahlgrenska Academy University of Gothenburg Gothenburg Sweden; ^4^ Department of Orthopaedics, Institute of Clinical Sciences, The Sahlgrenska Academy University of Gothenburg Gothenburg Sweden

**Keywords:** ACL reconstruction, anterior cruciate ligament, KOOS, Limb Symmetry Index, PASS, subjective knee function

## Abstract

**Purpose:**

To determine the proportion of patients and the factors associated with the achievement of a patient‐acceptable symptom state (PASS) 5 years after primary anterior cruciate ligament reconstruction (ACLR).

**Methods:**

Patients who underwent primary ACLR between 2005 and 2018 at Capio Artro Clinic, Stockholm, Sweden, were eligible for inclusion. The primary outcome was the achievement of a PASS on each Knee injury and Osteoarthritis Outcome Score (KOOS) subscale at the 5‐year follow‐up. Multivariable logistic regression analyses were used to assess associations with age, sex, time from injury to surgery, pre‐injury Tegner activity level, graft type, cartilage injury, meniscal resection or repair, and symmetrical 6‐month limb symmetry index (LSI ≥ 90%) in isokinetic extension and flexion strength and single‐leg‐hop performance.

**Results:**

A total of 2663 patients were analysed. The proportion of patients achieving a PASS varied between the KOOS subscales as follows: pain 64.8%; symptoms 93.8%; activities of daily living (ADL) 49.1%; sport and recreation (Sport&Rec) 65.4% and quality of life (QoL) 73.5%. Age ≥30 years was positively associated with PASS across all subscales. Female sex reduced the odds of achieving a PASS on the pain (OR 0.80; 95% confidence interval [CI] 0.65–0.99; *p* = 0.04), symptoms (OR 0.57; 95% CI 0.36–0.90; *p* = 0.02) and Sport&Rec (OR 0.73; CI 95% 0.59–0.90; *p* = 0.004) subscales. A surgical delay of ≥3 months was negatively associated with the achievement of a PASS on the QoL (OR 0.71; 95% CI 0.51–0.99; *p* = 0.04) subscale. Medial meniscus (MM) repair reduced the odds of achieving a PASS on the Sport&Rec (OR 0.61; 95% CI 0.41–0.92; *p* = 0.02) and QoL (OR 0.56; 95% CI 0.37–0.86; *p* = 0.01) subscales. Hamstring tendon (HT) autograft rather than bone‐patellar‐tendon‐bone autograft had increased odds of achieving a PASS on the Sport&Rec (OR 2.13; 95% CI 1.31–3.47; *p* = 0.002) subscale. Achieving a LSI ≥ 90% in isokinetic extension strength was associated with a PASS on ADL (OR 1.01; 95% CI 1.00–1.02; *p* = 0.02) and QoL (OR 1.01; 95% CI 1.00–1.02; *p* = 0.02) subscales, whereas an LSI ≥ 90% in single‐leg‐hop test was associated with a PASS on the symptoms (OR 1.03; 95% CI 1.01–1.05; *p* < 0.001), ADL (OR 1.01; 95% CI 1.00–1.02; *p* = 0.02), Sport&Rec (OR 1.01; 95% CI 1.01–1.02; *p* = 0.003), and QoL (OR 1.01; 95% CI 1.00–1.02; *p* = 0.01) subscales.

**Conclusion:**

The achievement of a PASS was over 64% on at least four out of five KOOS subscales 5 years after ACLR. Older age (≥ 30 years) was consistently associated with higher odds of achieving a PASS, while female sex was associated with lower odds. MM repair was associated with reduced odds of achieving a PASS on the Sport&Rec subscale, whereas the use of HT grafts was associated with higher odds on the same subscale. Symmetrical isokinetic extension strength and single‐leg‐hop test performance at 6 months were associated with higher odds of achieving a PASS; however, the effect sizes were small, suggesting limited clinical relevance.

**Level of Evidence:**

Level III.

**Abbreviations**
ACL: anterior cruciate ligament
ACLR: anterior cruciate ligament reconstruction
ADL: activities of daily living
BPTB: bone‐patellar tendon‐bone
CI: confidence interval
HT: hamstring tendon
KOOS: Knee Injury And Osteoarthritis Outcome Score
LM: lateral meniscus
LSI: Limb Symmetry Index
MM: medial meniscus
OR: odds ratio
PASS: patient acceptable symptom state
PROMs: patient reported outcome measures
QoL: quality of life
ROM: range of motion
SD: standard deviation
SE: standard error
SKLR: Swedish Knee Ligament Registry

## INTRODUCTION

Anterior cruciate ligament (ACL) injuries are common in young, active individuals [15], and ACL reconstruction (ACLR) is frequently performed to restore knee stability and allow return to sport [16, 34]. However, concomitant injuries and the risk of impaired long‐term outcomes highlight the importance of assessing knee function from the patient's perspective [5, 25, 32].

Patient‐reported outcome measures (PROMs) are widely recognised as essential tools for capturing the patient's perspective on treatment efficacy following orthopaedic procedures [36]. Among these, the Knee injury and Osteoarthritis Outcome Score (KOOS) [32] has become one of the most frequently used PROMs to evaluate patient‐perceived knee function after ACLR [12, 20, 21, 22]. Despite its broad use, interpretation of absolute postoperative KOOS scores remains complex, as clinician‐defined success does not always align with what patients consider a satisfactory knee function. To address this limitation, Muller et al. [27] established threshold values for the achievement of a Patient Acceptable Symptom State (PASS) for each KOOS subscale between 1 and 5 years following primary ACLR. These thresholds were determined based on patients' responses to the anchor question: ‘Taking into account all the activity you have during your daily life, your level of pain, and also your activity limitations and participation restrictions, do you consider the current state of your knee satisfactory?’

Incorporating PASS thresholds into KOOS interpretation offers an opportunity to move beyond statistical significance or clinician‐defined success and anchor outcomes in clinically meaningful benchmarks defined by the patients themselves.

Multiple factors may be associated with the likelihood of achieving a PASS following ACLR. However, current understanding of these factors remains limited. The considerable heterogeneity in preoperative, intraoperative, and postoperative patient characteristics complicates the ability of accurately predict which individuals will obtain a satisfactory outcome after primary ACLR. If modifiable factors are identified, targeted adjustments to the treatment strategy may enhance the likelihood of a satisfactory result. A comprehensive understanding of these factors thus provides an opportunity to individualise care and improve outcomes following primary ACLR. To date, there is a lack of studies that have evaluated the mid‐term rate of PASS achievement following primary ACLR in a large cohort. Moreover, a comprehensive analysis of patient‐related factors associated with the likelihood of achieving a PASS at mid‐term has yet to be presented.

The aim of this study was to determine the proportion of patients achieving a PASS 5 years after primary ACLR, and to identify preoperative, intraoperative, and postoperative factors associated with PASS achievement in a large patient cohort. It was hypothesised that age, sex, graft type, presence of concomitant meniscal surgery or cartilage injury, as well as recovery of symmetrical isokinetic extension or flexion strength at 6 months would be associated with the likelihood of achieving a PASS in the KOOS 5 years following ACLR.

## MATERIALS AND METHODS

Ethical approval for this study was granted by the Regional Ethics Committee at Karolinska Institute (Dnr 2016/1613‐31/2). The STrengthening the Reporting of OBservational studies in Epidemiology (STROBE) statement was used as guidance to report this study to increase scientific quality [13].

Patients who underwent primary ACLR using either hamstring tendon (HT) or bone‐patellar tendon‐bone (BPTB) autografts at Capio Artro Clinic, Stockholm, Sweden, between 2005 and 2018 were eligible for inclusion. Exclusion criteria were concomitant ligament injuries, contralateral ACL injury or reconstruction, as well as revision ACLR within 5 years from the primary procedure.

### Surgical technique and rehabilitation

All patients underwent primary ACLR using a single‐bundle autologous HT or BPTB graft. For procedures involving HT grafts, the semitendinosus tendon was primarily harvested and configured as a triple‐ or quadruple‐stranded graft. If the resulting graft was deemed insufficient in length or diameter (< 8 mm), the gracilis tendon was additionally harvested and incorporated. BPTB grafts were harvested as the central third of the patellar tendon, including two bone blocks. Femoral tunnels were created using an anteromedial portal technique. Graft fixation was standardised, with femoral fixation performed using an Endobutton device (Smith & Nephew, Andover, MA, USA) and tibial fixation achieved using either Ethibond no. 2 sutures (Ethicon, Sommerville, NJ, USA) tied over an AO bicortical screw with a washer, or with an interference screw. Meniscal repair was performed on both the medial meniscus (MM) and lateral meniscus (LM) using an all‐inside technique with Fast‐Fix suture anchor devices (Smith & Nephew, Andover, MA, USA) for tears located in the posterior and mid‐portions of the menisci. An outside‐in technique using PDS 0 (Ethicon, Sommerville, NJ, USA) was used for tears located in the anterior portion of the menisci.

All patients adhered to a standardised postoperative rehabilitation protocol. In cases of isolated ACLR or ACLR with combined meniscal resection, full weight‐bearing and unrestricted range of motion (ROM) were encouraged as tolerated. For patients who underwent concomitant meniscal repair, a hinged knee brace was prescribed for 6 weeks. ROM was progressively limited: 0°–30° during the first 2 weeks, 0°‐60° during Weeks 3 and 4, and 0°–90° during Weeks 5 and 6. Beginning in the seventh postoperative week, the brace was discontinued. Early rehabilitation emphasised restoration of ROM, reduction of joint swelling, and normalisation of gait pattern. The rehabilitation programme included joint and muscle flexibility exercises, balance and coordination training, and progressive strength training with an emphasis on the thigh musculature. Quadriceps strengthening was limited to closed kinetic chain exercises during the first three months postoperatively. Return to sports was permitted no earlier than six months after surgery and was based on the patient's recovery of muscle strength, coordination, hop performance, and the demands of their specific sport.

### Isokinetic strength and single‐leg‐hop test performance assessment

At six months postoperatively, patients underwent assessment of isokinetic strength and single‐leg‐hop test performance according to a standardised protocol. Bilateral concentric extension and flexion strength were evaluated at 90°/s using the Biodex System 3 dynamometer (Biodex Medical Systems, Shirley, NY, USA). Testing was conducted through a ROM from 90° to 10° of knee flexion, always beginning with the contralateral, uninjured limb. Prior to testing, patients completed a 10‐min warm‐up on o stationary cycling ergometer at low resistance. Standardised instructions were provided, and two to three practice trials were allowed. Each leg performed five maximal repetitions for both extension and flexion, and the peak torque values—the highest force generated—were recorded. Verbal encouragement was given throughout the test.

The single‐leg‐hop test was used to evaluate functional performance [30, 33]. Patients were instructed to stand on one leg, jump forward as far as possible, and land on the same leg while maintaining balance. A trial was considered successful if the landing was stable. The test was repeated if there was premature contact on the floor with the contralateral limb, loss of balance, or additional hops after landing. Prior to testing, patients received verbal instructions and were allowed unlimited practice trials to ensure confidence in performing the test. Three recorded trials were conducted for each leg, always beginning with the contralateral, uninjured side. Patients were given sufficient rest between attempts to minimise fatigue. The best distance achieved for each leg was recorded.

Symmetry in isokinetic extension and flexion strength, as well as in single‐leg‐hop test performance, was defined using the limb symmetry index (LSI). An LSI ≥ 90%, indicating that the involved limb performed at least 90% as well as the uninvolved limb, was considered the threshold for symmetrical function in each respective test [19, 29, 39].

### Data sources

Demographic variables, including age and sex, as well as clinical data such as time from injury to surgery, pre‐injury Tegner activity level [37], graft type, meniscal surgery (resection or repair), presence of cartilage injuries, and the results from the isokinetic extension and flexion strength tests and single‐leg‐hop test at 6 months postoperatively, were obtained from our clinic registry. The 5‐year KOOS subscale scores were obtained from the Swedish Knee Ligament Registry (SKLR) [38].

### Outcome

The primary outcome of this study was the proportion of patients achieving a PASS for each KOOS subscale 5 years after primary ACLR. The KOOS was developed to evaluate symptoms and subjective knee function following knee injury. It comprises five independent subscales: Pain, Symptoms, Activities of Daily Living (ADL), Sport and Recreation and Knee‐Related Quality of Life (QoL). According to Roos et al. [32], the Sport and Recreation and QoL subscales are the most responsive KOOS domains in detecting postoperative changes following ACLR. Each subscale includes between 4 and 17 items, with responses recorded on a 5‐point Likert scale ranging from 0 (no problems) to 4 (extreme problems). Subscale scores are transformed to a 0–100 scale, where 0 represents “extreme knee problems” and 100 representing “no knee problems”. It is recommended that each KOOS subscale be evaluated independently, as they represent distinct dimensions of knee function and should not be aggregated into a single composite score [32]. PASS achievement for each KOOS subscale was determined using the threshold values established by Muller et al. [28]. The corresponding cut‐off scores were Pain ≥ 88.9, Symptoms ≥ 57.1, ADL = 100, Sport and Recreation ≥ 75.0, QoL ≥ 62.5.

### Statistical analysis

The Statistical Package for Social Sciences, SPSS (Version 27.0, IBM Corp., Armonk, New York, USA) was used for the statistics. All variables were summarised using standard descriptive statistics, including means with standard deviations for continuous variables and frequencies for categorical variables. The normality of data distributions was assessed visually. To evaluate differences between patients included in the 5‐year KOOS follow‐up and those lost to follow‐up, the Pearson's Chi‐square test and the Student's t‐test were used for categorical and continuous variables, respectively. The achievement of a PASS for each KOOS subscale was analysed separately as the primary outcome. Multivariable logistic regression analyses were used to identify factors associated with PASS achievement. Independent variables included age (≥30 years vs. <30 years), sex (female vs. male), time from injury to surgery (> 3 months vs. ≤3 months), pre‐injury Tegner activity level (high ≥6 vs. low <6), graft type (HT vs. BPTB autograft), MM resection, MM repair, LM resection, LM repair, presence of cartilage injury, and recovery of 6‐month symmetrical (LSI ≥ 90%) isokinetic extension strength, flexion strength, and single‐leg‐hop performance. Age was dichotomised into unbiased classes close to the median (30 years). Time from injury to surgery was dichotomised at 3 months, as this represents the recommended period for patients in our clinical practice to undergo a trial of conservative treatment with rehabilitation, when clinically appropriate. Each KOOS subscale PASS outcome served as a separate dependent variable. Logistic regression analysis results were reported as odds ratios (ORs) with corresponding 95% confidence intervals (CIs). The level of significance in all analyses was 5% (two‐tailed).

## RESULTS

A total of 7134 patients who underwent primary ACLR were screened for inclusion. Following the exclusion of patients with concomitant ligament injuries (*n* = 501), contralateral ACL injury or reconstruction (*n* = 308), as well as revision ACLR within 5 years of the primary procedure (*n* = 375), 5950 patients remained eligible for inclusion. Of these, 3287 patients (55.2%) lacked KOOS data at the 5‐year follow‐up and were therefore excluded from the outcome analysis. A total of 2663 patients met the inclusion criteria and had complete 5‐year postoperative KOOS data available for all subscales. The follow‐up rate for 5‐year KOOS outcomes was 44.8% (2663 out of 5950 patients). A comparison between patients included in the 5‐year follow‐up and those lost to follow‐up is presented in Table 1. Patients without 5‐year KOOS data were significantly younger than those with available data (27.9 vs. 31.3 years; *p* < 0.001). Female representation was significantly higher in the included cohort compared to those without 5‐year KOOS data (51.4% vs. 39.8%; *p* < 0.001). Patients without 5‐year KOOS data had a significantly greater proportion of MM repairs (8.5% vs. 6.6%; *p* = 0.01), whereas the prevalence of cartilage injuries was significantly higher among those included in the 5‐year follow‐up (19.8% vs. 17.3%; *p* = 0.01). No other significant differences were observed between the groups (Table 1).

**Table 1:** Patient characteristics and dropout analysis.

	Included cohort (*n* = 2663)	No 5‐year KOOS data (*n* = 3287)	*p*‐value
Pre‐operative factors			
Age at surgery, years, mean ± SD	31.3 ± 11.5	27.9 ± 10.1	<0.001
Age younger than 30 years	20.9 ± 5.0; 1253 (47.1)	21.0 ± 4.7; 1973 (60.1)	
Age 30 years or older	40.5 ± 6.9; 1410 (52.9)	38.3 ± 6.4; 1312 (39.9)	
Sex			<0.001
Male	1293 (48.6)	1980 (60.2)	
Female	1370 (51.4)	1307 (39.8)	
Time from injury to surgery, months, mean ± SD	21.8 ± 45.4	20.6 ± 38.1	n.s.
≤3 months	397 (15.1)	522 (16.1)	
>3 months	2236 (84.9) *n* = 2633	2723 (83.9) *n* = 3245	
Pre‐injury Tegner activity level, median (range)	7 (0–10)	7 (0–10)	n.s.
High, ≥6	1971 (84.1)	2409 (85.9)	
Low, <6	373 (15.9) *n* = 2344	397 (14.1) *n* = 2806	
Intra‐operative factors			
Graft type			n.s.
HT autograft	2514 (94.4)	3085 (93.9)	
BPTB autograft	149 (5.6)	202 (6.1)	
Medial meniscus surgery			
Resection	420 (15.8)	488 (14.8)	n.s
Repair	176 (6.6)	281 (8.5)	0.01
Lateral meniscus surgery			
Resection	390 (14.6)	542 (16.5)	n.s.
Repair	107 (4.0)	157 (4.8)	n.s.
Cartilage injury			0.01
Yes	528 (19.8)	568 (17.3)	
No	2135 (80.2)	2719 (82.7)	
Post‐operative factors (6 months)			
Isokinetic extension strength			n.s.
LSI, mean ± SD	84.0 ± 16.0	84.0 ± 16.0	
LSI ≥ 90%	771 (33.2)	908 (34.9)	
LSI < 90%	1550 (66.8) *n* = 2321	1690 (65.1) *n* = 2598	
Isokinetic flexion strength			n.s.
LSI, mean ± SD	90.0 ± 16.0	90.0 ± 22.0	
LSI ≥ 90%	1116 (48.2)	1216 (46.9)	
LSI < 90%	1201 (51.8) *n* = 2317	1379 (53.1) *n* = 2595	
Single‐leg‐hop test			
LSI, mean ± SD	92.0 ± 14.0	92.0 ± 13.0	n.s.
LSI ≥ 90%	1286 (66.1)	1404 (67.9)	
LSI < 90%	661 (33.9) *n* = 1947	664 (32.1) *n* = 2068	

*Note*: Data are reported as *n* (%) unless otherwise indicated.

Abbreviations: BPTB, bone‐patellar tendon bone; HT, hamstring tendon; KOOS, Knee Injury and Osteoarthritis Outcome Score; LSI, limb symmetry index; SD, standard deviation.

Mean KOOS scores at the 5‐year follow‐up and PASS rates for each dichotomised patient subgroup within the included cohort are presented in Table 2. The proportion of patients achieving a PASS varied between the KOOS subscales as follows: Pain 64.8%; symptoms 93.8%; ADL 49.1%; sport and recreation 65.4% and QoL 73.5% (Table 2).

**Table 2:** Mean ± SD (PASS %) KOOS values for each dichotomised patient group in the included cohort (*n* = 2663) at the 5‐year follow up.

	Pain (≥88.9)	Symptoms (≥57.1)	ADL (=100)	Sports and recreation (≥75.0)	QoL (≥62.5)
Pre‐operative factors					
Age at surgery					
Age <30 years	88.6 ± 13.7 (60.2)	82.4 ± 16.2 (92.2)	94.6 ± 10.3 (46.4)	74.8 ± 23.9 (63.2)	68.9 ± 22.5 (69.1)
Age ≥ 30 years	90.7 ± 12.9 (68.9)	86.7 ± 15.1 (95.2)	94.7 ± 10.4 (51.6)	77.0 ± 24.0 (67.4)	74.7 ± 21.8 (77.4)
Sex					
Male	90.7 ± 12.7 (68.0)	85.5 ± 15.3 (94.7)	94.7 ± 10.3 (52.2)	78.2 ± 22.7 (69.4)	73.2 ± 22.2 (75.1)
Female	88.8 ± 13.8 (61.8)	83.9 ± 16.2 (92.9)	94.3 ± 10.3 (46.2)	73.8 ± 24.9 (61.7)	70.8 ± 22.3 (72.0)
Time from injury to surgery					
≤3 months	91.1 ± 12.2 (69.3)	85.4 ± 15.2 (93.7)	96.0 ± 8.4 (52.9)	78.2 ± 23.3 (69.4)	75.3 ± 21.9 (76.8)
>3 months	89.4 ± 13.5 (64.1)	84.6 ± 15.9 (93.8)	94.4 ± 10.6 (48.3)	75.5 ± 24.0 (64.8)	71.4 ± 22.2 (73.1)
Pre‐injury Tegner activity level					
High, ≥6	90.0 ± 13.1 (65.6)	84.7 ± 15.8 (93.7)	94.9 ± 9.9 (50.2)	76.4 ± 23.4 (65.4)	72.1 ± 22.1 (73.8)
Low, <6	88.0 ± 14.7 (60.3)	84.4 ± 16.0 (93.3)	93.2 ± 12.2 (44.0)	73.8 ± 26.1 (64.5)	71.0 ± 22.7 (73.1)
Intra‐operative factors					
Graft type					
HT autograft	89.8 ± 13.4 (65.2)	84.8 ± 15.8 (93.8)	94.6 ± 10.4 (49.6)	76.2 ± 24.1 (66.0)	72.1 ± 22.3 (73.8)
BPTB autograft	88.7 ± 12.5 (58.4)	83.1 ± 16.1 (93.3)	94.4 ± 9.7 (40.9)	72.3 ± 22.3 (56.1)	69.9 ± 21.9 (68.9)
Medial meniscus surgery					
Resection	89.5 ± 13.9 (64.8)	85.0 ± 16.0 (92.4)	94.0 ± 11.2 (50.7)	75.3 ± 24.5 (64.2)	71.2 ± 22.7 (71.8)
Repair	87.6 ± 14.5 (54.5)	81.4 ± 16.2 (89.2)	93.6 ± 11.1 (41.5)	71.2 ± 26.2 (57.7)	67.3 ± 22.4 (63.6)
Lateral meniscus surgery					
Resection	89.3 ± 14.3 (64.6)	83.0 ± 16.9 (91.0)	93.7 ± 12.2 (47.9)	74.7 ± 24.5 (63.2)	70.6 ± 22.8 (71.3)
Repair	90.6 ± 11.5 (65.4)	84.7 ± 14.6 (95.3)	96.1 ± 8.2 (51.4)	76.5 ± 22.5 (69.2)	73.7 ± 19.4 (76.6)
Cartilage injury					
Yes	88.7 ± 13.4 (59.3)	83.7 ± 16.6 (93.0)	93.6 ± 10.9 (44.7)	72.2 ± 26.1 (59.6)	70.6 ± 23.0 (72.1)
No	90.0 ± 13.3 (66.1)	84.9 ± 15.6 (94.0)	94.9 ± 10.2 (50.2)	76.9 ± 23.3 (66.9)	72.3 ± 22.1 (73.9)
Post‐operative factors (6 months)					
Isokinetic extension strength					
LSI ≥ 90%	91.9 ± 11.6 (70.6)	86.8 ± 14.6 (96.0)	96.2 ± 8.7 (56.7)	79.5 ± 22.4 (71.0)	76.0 ± 20.6 (80.4)
LSI < 90%	89.5 ± 13.3 (65.1)	84.5 ± 13.8 (93.1)	94.5 ± 10.2 (47.4)	75.9 ± 23.3 (65.2)	71.3 ± 22.2 (72.6)
Isokinetic flexion strength					
LSI ≥ 90%	91.4 ± 11.7 (70.9)	86.3 ± 14.8 (94.6)	95.8 ± 8.8 (52.9)	78.9 ± 22.0 (70.8)	75.0 ± 21.0 (78.1)
LSI < 90%	89.2 ± 13.7 (63.2)	84.3 ± 16.0 (93.5)	94.4 ± 10.5 (48.2)	75.4 ± 23.9 (63.8)	70.9 ± 22.3 (72.5)
Single‐leg‐hop test					
LSI ≥ 90%	91.4 ± 12.0 (69.8)	86.3 ± 14.4 (95.7)	96.0 ± 8.9 (55.1)	79.8 ± 21.6 (71.3)	74.7 ± 21.2 (77.5)
LSI < 90%	89.4 ± 13.3 (65.4)	84.3 ± 16.5 (92.4)	94.3 ± 10.1 (46.0)	75.2 ± 23.2 (63.8)	70.9 ± 22.1 (72.5)

*Note*: The threshold values of PASS for each subscale are presented in the first row.

Abbreviations: ADL, activities of daily living; BPTB, bone‐patellar tendon bone; HT, hamstring tendon; KOOS, Knee injury and Osteoarthritis Outcome Score; LSI, limb symmetry index; PASS, patient‐acceptable symptom state; QoL, Quality of Life; SD, standard deviation.

Age ≥30 years was associated with increased odds of achieving a PASS across all KOOS subscales. Female sex was associated with lower odds of achieving a PASS on the Pain, Symptoms, and Sport and Recreation subscales. A surgical delay of more than 3 months was negatively associated with PASS achievement on the QoL subscale. Patients who underwent MM repair had reduced odds of achieving a PASS on the Sport and Recreation and QoL subscales. The use of a HT autograft, as compared to a BPTB autograft, was associated with higher odds of achieving a PASS on the Sport and Recreation subscale. Achieving an LSI ≥ 90% in isokinetic extension strength at 6 months was positively associated with PASS achievement on the ADL and QoL subscales, whereas an LSI ≥ 90% in single‐leg‐hop performance was associated with a higher likelihood of achieving a PASS on the Symptoms, ADL, Sport and Recreation, and QoL subscales. No additional variables were found to be significantly associated with the achievement of a PASS in the remaining KOOS subscales at the 5‐year follow‐up after ACLR (Tables 3, 4, 5, 6, 7).

**Table 3:** Factors affecting the achievement of a PASS on the KOOS pain (≥ 88.9) subscale.

Factor	Regression coefficient (ß)	SE	Wald	OR (95% CI)	*p*‐value
Pre‐operative					
Age ≥30 years	0.74	0.12	41.01	2.09 (1.67–2.62)	<0.001
Female sex	−0.23	0.11	4.30	0.80 (0.65–0.99)	0.04
Delayed (>3 months) ACLR	−0.25	0.15	2.67	0.78 (0.58–1.05)	n.s.
Pre‐injury Tegner activity level ≥6	0.05	0.16	0.11	1.05 (0.78–1.43)	n.s.
Intra‐operative					
HT autograft	0.31	0.26	1.49	1.37 (0.83–2.25)	n.s.
MM resection	−0.04	0.15	0.07	0.96 (0.71–1.30)	n.s.
MM repair	−0.38	0.21	3.33	0.68 (0.45–1.03)	n.s.
LM resection	0.11	0.15	0.52	1.12 (0.83–1.51)	n.s.
LM repair	−0.29	0.28	1.08	0.75 (0.43–1.30)	n.s.
Cartilage injury	−0.23	0.14	2.64	0.80 (0.60–1.05)	n.s.
Post‐operative (6 months)					
Extension strength LSI ≥ 90%	0.004	0.004	0.97	1.00 (1.00–1.01)	n.s.
Flexion strength LSI ≥ 90%	0.003	0.004	0.78	1.00 (1.00–1.01)	n.s.
Single‐leg‐hop test LSI ≥ 90%	0.006	0.01	1.68	1.01 (1.00–1.02)	n.s.

*Note*: ACLR, anterior cruciate ligament reconstruction; CI, confidence interval; HT, hamstring tendon; KOOS, Knee injury and Osteoarthritis Outcome Score; LM, lateral meniscus; LSI, limb symmetry index; MM, medial meniscus; OR, odds ratio; PASS, Patient‐acceptable symptom state; SE, standard error.

**Table 4:** Factors affecting the achievement of a PASS on the KOOS symptoms (≥ 57.1) subscale.

Factor	Regression coefficient (ß)	SE	Wald	OR (95% CI)	*p‐*value
Pre‐operative					
Age ≥30 years	1.13	0.26	18.60	3.08 (1.85–5.14)	<0.001
Female sex	−0.56	0.24	5.71	0.57 (0.36–0.90)	0.02
Delayed (>3 months) ACLR	0.05	0.30	0.02	1.05 (0.58–1.87)	n.s.
Pre‐injury Tegner activity level ≥6	−0.19	0.34	0.29	0.83 (0.43–1.62)	n.s.
Intra‐operative					
HT autograft	−0.15	0.55	0.07	0.86 (0.29–2.54)	n.s.
MM resection	−0.36	0.30	1.38	0.70 (0.39–1.27)	n.s.
MM repair	−0.44	0.36	1.48	0.64 (0.32–1.31)	n.s.
LM resection	−0.21	0.29	0.50	0.81 (0.46–1.45)	n.s.
LM repair	−0.14	0.54	0.07	0.87 (0.30–2.51)	n.s.
Cartilage injury	−0.19	0.29	0.44	0.83 (0.47–1.45)	n.s.
Post‐operative (6 months)					
Extension strength LSI ≥ 90%	0.004	0.01	0.18	1.00 (0.99–1.02)	n.s.
Flexion strength LSI ≥ 90%	−0.001	0.01	0.02	1.00 (0.98–1.02)	n.s.
Single‐leg‐hop test LSI ≥ 90%	0.03	0.01	12.88	1.03 (1.01–1.05)	<0.001

Abbreviations: ACLR, anterior cruciate ligament reconstruction; CI, confidence interval; HT, hamstring tendon; KOOS, Knee injury and Osteoarthritis Outcome Score; LM, lateral meniscus; LSI, limb symmetry index; MM, medial meniscus; OR, odds ratio; PASS, Patient‐acceptable symptom state; SE, standard error.

**Table 5:** Factors affecting the achievement of a PASS on the KOOS ADL (=100.0) subscale.

Factor	Regression coefficient (ß)	SE	Wald	OR (95% CI)	*p*‐value
Pre‐operative					
Age ≥30 years	0.52	0.11	24.06	1.68 (1.37–2.07)	<0.001
Female sex	−0.18	0.10	3.24	0.83 (0.68–1.02)	n.s.
Delayed (>3 months) ACLR	−0.14	0.14	1.06	0.87 (0.66–1.14)	n.s.
Pre‐injury Tegner activity level ≥6	0.07	0.15	0.24	1.07 (0.81–1.43)	n.s.
Intra‐operative					
HT autograft	0.32	0.25	1.67	1.38 (0.85–2.23)	n.s.
MM resection	0.08	0.14	0.29	1.08 (0.82–1.42)	n.s.
MM repair	−0.39	0.21	3.43	0.68 (0.45–1.02)	n.s.
LM resection	−0.06	0.14	0.18	0.94 (0.72–1.24)	n.s.
LM repair	0.01	0.28	0.002	1.01 (0.59–1.75)	n.s.
Cartilage injury	−0.16	0.13	1.54	0.85 (0.66–1.10)	n.s.
Post‐operative (6 months)					
Extension strength LSI ≥ 90%	0.01	0.004	5.57	1.01 (1.00–1.02)	0.02
Flexion strength LSI ≥ 90%	−0.002	0.003	0.33	1.00 (0.99–1.01)	n.s.
Single‐leg‐hop test LSI ≥ 90%	0.01	0.004	5.15	1.01 (1.00–1.02)	0.02

Abbreviations: ACLR, anterior cruciate ligament reconstruction; ADL, activities of daily living; CI, confidence interval; HT, hamstring tendon; KOOS, Knee injury and Osteoarthritis Outcome Score; LM, lateral meniscus; LSI, limb symmetry index; MM, medial meniscus; OR, odds ratio; PASS, Patient‐acceptable symptom state; SE, standard error.

**Table 6:** Factors affecting the achievement of a PASS on the KOOS sports and recreation ( ≥ 75.0) subscale.

Factor	Regression coefficient (ß)	SE	Wald	OR (95% CI)	*p*‐value
Pre‐operative					
Age ≥30 years	0.49	0.11	18.35	1.63 (1.31–2.04)	<0.001
Female sex	−0.32	0.11	8.40	0.73 (0.59–0.90)	0.004
Delayed (>3 months) ACLR	−0.16	0.15	1.05	0.86 (0.64–1.15)	n.s.
Pre‐injury Tegner activity level ≥6	−0.12	0.16	0.52	0.89 (0.65–1.22)	n.s.
Intra‐operative					
HT autograft	0.76	0.25	9.17	2.13 (1.31–3.47)	0.002
MM resection	0.03	0.15	0.05	1.03 (0.76–1.40)	n.s.
MM repair	−0.50	0.21	5.69	0.61 (0.41–0.92)	0.02
LM resection	−0.10	0.15	0.43	0.91 (0.68–1.22)	n.s.
LM repair	−0.13	0.29	0.21	0.88 (0.50–1.55)	n.s.
Cartilage injury	−0.17	0.14	1.49	0.84 (0.64–1.11)	n.s.
Post‐operative (6 months)					
Extension strength LSI ≥ 90%	0.002	0.004	0.25	1.00 (0.99–1.01)	n.s.
Flexion strength LSI ≥ 90%	0.01	0.004	3.82	1.01 (1.00–1.02)	n.s.
Single‐leg‐hop test LSI ≥ 90%	0.01	0.01	8.76	1.01(1.01–1.02)	0.003

Abbreviations: ACLR, anterior cruciate ligament reconstruction; CI, confidence interval; HT, hamstring tendon; KOOS, Knee injury and Osteoarthritis Outcome Score; LM, lateral meniscus; LSI, limb symmetry index; MM, medial meniscus; OR, odds ratio; PASS, Patient‐acceptable symptom state; SE, standard error.

**Table 7:** Factors affecting the achievement of a PASS on the KOOS QoL ( ≥ 62.5) subscale.

Factor	Regression coefficient (ß)	SE	Wald	OR (95% CI)	*p*‐value
Pre‐operative					
Age ≥30 years	0.88	0.13	47.08	2.42 (1.88–3.11)	<0.001
Female sex	−0.08	0.12	0.41	0.93 (0.73–1.17)	n.s.
Delayed (>3 months) ACLR	−0.35	0.17	4.11	0.71 (0.51–0.99)	0.04
Pre‐injury Tegner activity level ≥6	−0.27	0.18	2.10	0.77 (0.54–1.10)	n.s.
Intra‐operative					
HT autograft	0.43	0.27	2.60	1.54 (0.91–2.60)	n.s.
MM resection	−0.14	0.17	0.70	0.87 (0.63–1.21)	n.s.
MM repair	−0.58	0.22	7.20	0.56 (0.37–0.86)	0.01
LM resection	−0.04	0.16	0.06	0.96 (0.70–1.32)	n.s.
LM repair	−0.08	0.31	0.07	0.92 (0.50–1.70)	n.s.
Cartilage injury	0.02	0.16	0.01	1.02 (0.74–1.39)	n.s.
Post‐operative (6 months)					
Extension strength LSI ≥ 90%	0.01	0.01	5.93	1.01 (1.00–1.02)	0.02
Flexion strength LSI ≥ 90%	0.001	0.004	0.12	1.00 (0.99–1.01)	n.s.
Single‐leg‐hop test LSI ≥ 90%	0.01	0.01	7.64	1.01 (1.00–1.02)	0.01

Abbreviations: ACLR, anterior cruciate ligament reconstruction; CI, confidence interval; HT, hamstring tendon; KOOS, Knee injury and Osteoarthritis Outcome Score; LM, lateral meniscus; LSI, limb symmetry index; MM, medial meniscus; OR, odds ratio; QoL, Quality of Living; PASS, Patient‐acceptable symptom state; SE, standard error.

## DISCUSSION

The most important finding of this study was the notable variation in the proportion of patients achieving a PASS across the KOOS subscales 5 years after ACLR. The rates ranged from 49.1% for the ADL subscale to 93.8% for the Symptoms subscale. Importantly, the achievement of a PASS was over 64% on at least four out of five KOOS subscales, indicating that the majority of patients perceived a satisfactory knee function in several key domains of recovery. Age ≥ 30 years was consistently associated with a higher likelihood of achieving a PASS across all KOOS subscales. In contrast, female sex was associated to reduced odds of achieving a PASS on the pain, symptoms, and sports and recreation subscales. Furthermore, achieving symmetrical (LSI ≥ 90%) isokinetic extension strength and single‐leg‐hop test performance at six months postoperatively was positively associated with the achievement of a PASS on the ADL and QoL subscales, and on the Symptoms, ADL, Sports and Recreation and QoL subscales, respectively.

The influence of age on subjective knee function following primary ACLR has previously been examined, although findings across studies have been inconsistent [1, 9, 18, 26, 35, 40]. Some studies found no significant association between age and KOOS outcomes [1, 26] while others found better outcomes in either younger or older patients [18, 35, 40]. Notably, Cristiani et al. [9], in a large cohort study, demonstrated that patients aged ≥ 30 years had superior KOOS scores and PASS achievement across all subscales at two‐year follow‐up. Our findings are in line with those of Cristiani et al. [9], as we found that age ≥ 30 years was consistently associated with greater odds of PASS achievement across all KOOS subscales at 5 years after ACLR. Younger patients may have greater expectations regarding their postoperative knee function and are less likely to accept residual symptoms or limitations as satisfactory [9].

Female sex was independently associated with decreased odds of achieving a PASS on the Pain, Symptoms, and Sports and Recreation subscales. These findings are consistent with previous research. For instance, a recent systematic review showed that female patients report inferior PROMs after ACLR [4]. Similarly, a recent large cohort registry study [9] identified female sex as a negative predictor of PASS achievement on the Pain, ADL and Sports and Recreation KOOS subscales 2 years after ACLR. Sex‐related disparities in KOOS and PASS outcomes following ACLR are likely multifactorial. Research suggest that females tend to report greater pain, lower psychological readiness, and reduced confidence in knee function compared to men [2, 14].

Longer (>3 months) time interval from injury to surgery was associated with decreased odds of achieving a PASS in the QoL subscale. Patients who undergo delayed ACLR are exposed to prolonged periods of knee instability, which can lead to decreased activity levels, psychological distress, and a diminished sense of joint trust—all of which are captured within the QoL subscale [15].

In the present study, the use of HT autografts was associated with a higher likelihood of achieving a PASS on the Sports and Recreation subscale at the 5‐year follow‐up compared to BPTB autografts. This difference could be explained by the “donor‐site morbidity” associated with the BPTB autograft [11, 43].

Interestingly, we were unable to find any significant association between MM or LM resection and the likelihood of achieving a PASS on any KOOS subscale at the 5‐year follow‐up. While this suggests that partial meniscectomy may not influence mid‐term patient‐reported outcomes, prior research has demonstrated that meniscal tissue loss can adversely affect long‐term knee function and increase the risk of degenerative changes following ACLR [3, 6, 7, 42]. It is possible that the negative consequences of meniscal resection require a longer duration to manifest and may become more apparent beyond the 5‐year postoperative period, once cumulative joint loading and cartilage wear progress [24].

Notably, MM repair was associated with reduced odds of achieving a PASS on the Sports and Recreation and QoL subscales, whereas LM repair did not demonstrate a significant association with PASS outcomes at the 5‐year follow‐up. Previous literature has shown that failure rates are higher for MM repair than for LM repair [10, 28], which may explain the observed findings. A recent large cohort study [10] demonstrated that patients with failed meniscal repair performed concurrently with ACLR exhibit significantly lower scores across all KOOS subscales at one year and on the Symptoms subscale at two years postoperatively. On the other hand, patients with successful MM repair did not demonstrate inferior outcomes compared to those undergoing isolated ACLR. Given that our analysis did not differentiate between successful and failed meniscal repairs, it is plausible that the MM repair group included a subset of patients with failed repairs, which may have adversely affected their subjective knee function as captured by the KOOS.

Restoration of symmetrical isokinetic extension and flexion strength and single‐leg‐hop performance is considered crucial before resuming sports activities following ACLR. Disparities in muscular strength and functional capacity are recognised contributors to an increased risk of graft failure and subsequent knee injuries [17, 23, 39]. Interestingly, in the present study, achieving an LSI ≥ 90% in isokinetic extension strength at 6 months increased the odds of achieving a PASS in the ADL and QoL subscales, while an LSI ≥ 90% in single‐leg‐hop test performance at 6 months increased the odds of achieving a PASS in the Symptoms, ADL, Sports and Recreation, and QoL subscales 5 years after primary ACLR. However, although these associations reached statistical significance, the observed ORs were close to 1.0, indicating minimal effect sizes. This likely reflects the influence of the large sample size, which may detect small statistical effects of limited clinical importance. In contrast, a large cohort study [9] which evaluated the association between symmetrical (LSI ≥ 90%) extension and flexion strength as well as single‐leg‐hop test performance at 6 months and the likelihood of achieving a PASS 2 years after primary ACLR reported higher effect sizes than those observed in the present study. This suggests that while muscle strength symmetry plays an important role on the achievement of a PASS 2 years after surgery, its clinical relevance in predicting patient‐perceived knee function at mid‐term follow‐up is limited. A possible explanation is that patients undergo lifestyle changes in the years following surgery. In our cohort, with a mean age of 31 at the time of surgery and 36 at follow‐up, increasing age may be associated with reduced physical activity and less frequent training. As a result, the influence of early postoperative symmetry on patient‐reported outcomes may diminish over time, making the distinction between those with and without symmetrical LSI less pronounced at 5 years [2].

One of the main strengths of this study is the large sample size (*n* = 2663), which enabled robust logistic regression analyses and the investigation of a wide range of factors potentially associated with PASS achievement after primary ACLR. The cohort was heterogeneous in terms of age, injury‐to‐surgery interval, pre‐injury activity level, and concomitant meniscal interventions, thereby improving the external validity of the findings. All patients were managed within a single healthcare setting, where surgical techniques, rehabilitation protocols, and follow‐up assessments followed standardised routines, reducing methodological variability. The use of PASS thresholds proposed by Muller et al. [27] allowed for a clinically meaningful interpretation of KOOS outcomes. Given the demographic and clinical similarities between this study population and that of Muller et al. [27], the application of these thresholds is considered appropriate. Reaching a PASS has been reported to align with the patient's perception of acceptable symptom state, serving as an important indicator of perceived treatment success.

The primary limitation of this study is the suboptimal follow‐up rate, with 44.8% of patients completing the KOOS questionnaires 5 years postoperatively. However, this rate is comparable to other large cohort registry studies in the field of ACLR [8, 9]. Despite this follow‐up rate, the absolute number of patients respondents remains very high, ensuring both strong statistical power and high generalisability of our findings. Notably, patients lost to follow‐up were generally younger and included a higher proportion of males compared to the analysed cohort. This pattern is consistent with the findings reported by Reinholdsson et al. [31] and may contribute to a selection bias, potentially limiting the generalisability of the results. Another limitation is the lack of detailed information regarding the size and severity of cartilage lesions as well as the extent and location of meniscal resections. Prior research has demonstrated that full‐thickness cartilage defects are associated with worse KOOS outcomes compared to partial‐thickness lesions five years after ACLR [41]. In our study, the binary classification of cartilage injury (present/absent) may have introduced residual confounding in the analysis of factors associated with achieving a PASS. A similar limitation applies to meniscal resections, for which no data were available on the resection extent or anatomical location. It is plausible that more extensive resections would have a greater negative impact on PROMs at follow‐up.

## CONCLUSION

The achievement of a PASS was over 64% on at least four out of five KOOS subscales 5 years after ACLR. Older age (≥30 years) was consistently associated with higher odds of achieving a PASS, while female sex was associated with lower odds. MM repair was associated with reduced odds of achieving a PASS on the Sport&Rec subscale, whereas the use of HT grafts was associated with higher odds of achieving a PASS on the same subscale. Symmetrical isokinetic extension strength and single‐leg‐hop test performance at 6 months were associated with higher odds of achieving a PASS; however, the effect sizes were small, suggesting limited clinical relevance.

## AUTHOR CONTRIBUTIONS

**Firathan Koca**: Data curation; methodology; and writing. **Christoffer von Essen**, **Eric Hamrin Senorski**, **Kristian Samuelsson**, and **Anders Stålman**: Methodology and critical review of the manuscript. **Riccardo Cristiani**: Conceptualisation; methodology; and critical review of the manuscript. All authors have given final approval of the version to be published.

## CONFLICT OF INTEREST STATEMENT

The authors declare no conflicts of interest.

## ETHICS STATEMENT

Ethical approval for this study was obtained from the regional ethics committee, Karolinska Institutet, Diarenumber 2016/1613‐31/32. No consent to participate is requested for registry studies.

## Data Availability

The data that support the findings of this study are available from the corresponding author upon reasonable request.
